# Certified high-efficiency “large-area” perovskite solar module for Fresnel lens-based concentrated photovoltaics

**DOI:** 10.1016/j.isci.2023.106079

**Published:** 2023-02-02

**Authors:** Anurag Roy, Bin Ding, Maria Khalid, Mussad Alzahrani, Yong Ding, Asif A. Tahir, Senthilarasu Sundaram, Sachin Kinge, Abdullah M. Asiri, Andre Slonopas, Paul J. Dyson, Mohammad Khaja Nazeeruddin, Tapas K. Mallick

**Affiliations:** 1 Solar Energy Resaerch Group, Environment and Sustainability Institute, Faculty of Environment, Science and Economy, University of Exeter, Penryn Campus, Penryn TR10 9FE, UK; 2 Institute of Chemical Sciences and Engineering, École Polytechnique Fédérale de Lausanne Valais Wallis, 1951 Sion, Switzerland; 3 Mechanical and Energy Engineering Department, Imam Abdulrahman Bin Faisal University, Dammam 34212, Saudi Arabia; 4 State Key Laboratory of Alternate Electrical Power System with Renewable Energy Sources, North China Electric Power University, Beijing 102206, People’s Republic of China; 5 Cybersecurity and Systems Engineering, School of Computing, Engineering and the Built Environment, Edinburgh Napier University, Merchiston Campus, Edinburgh EH10 5DT, UK; 6 Toyota Motor Europe, Materials Engineering Division, Hoge Wei 33, Zaventem 1820, Belgium; 7 Center of Excellence for Advanced Materials Research (CEAMR), King Abdulaziz University, P.O. Box 80203, Jeddah 21589, Saudi Arabia; 8 Johns Hopkins University, Whiting School of Engineering, 3400 N Charles Street, Baltimore, MD 21218, USA

**Keywords:** Energy systems, Optical property, Energy materials

## Abstract

The future of energy generation is well in tune with the critical needs of the global economy, leading to more green innovations and emissions-abatement technologies. Introducing concentrated photovoltaics (CPVs) is one of the most promising technologies owing to its high photo-conversion efficiency. Although most researchers use silicon and cadmium telluride for CPV, we investigate the potential in nascent technologies, such as perovskite solar cell (PSC). This work constitutes a preliminary investigation into a “large-area” PSC module under a Fresnel lens (FL) with a “refractive optical concentrator-silicon-on-glass” base to minimize the PV performance and scalability trade-off concerning the PSCs. The FL-PSC system measured the solar current-voltage characteristics in variable lens-to-cell distances and illuminations. The PSC module temperature was systematically studied using the COMSOL transient heat transfer mechanism. The FL-based technique for “large-area” PSC architectures is a promising technology that further facilitates the potential for commercialization.

## Highlights

• PSC modules with an aperture area of 28.58 cm^2^ secured a certified efficiency of ∼21%
• Fresnel lens-based higher concentrated photovoltaics integrated with PSC
• PV performance was evaluated under various solar irradiance and focal lengths
• Heat stress and thermo-kinetic simulation were performed using the COMSOL modeling

## Graphical abstract

**Figure:**
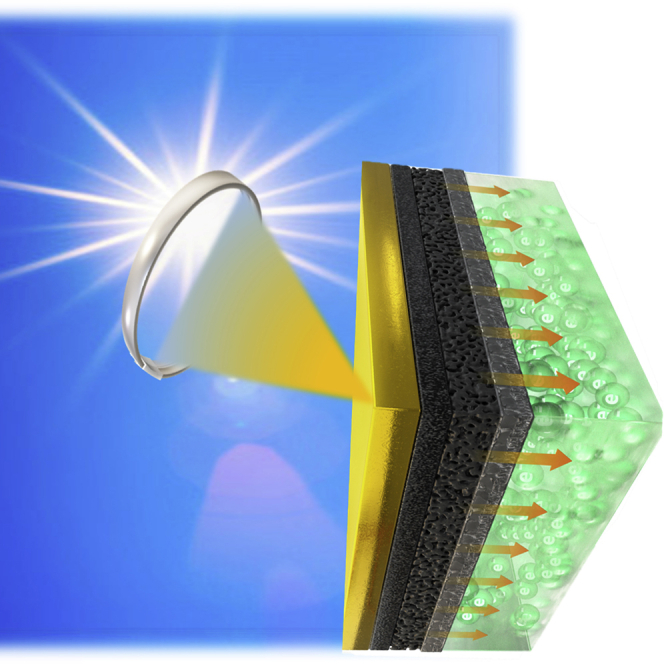

## Introduction

Alternative zero-emission renewable energy sources are becoming increasingly essential to support worldwide energy needs and to tackle the fast depletion of fossil fuels and their severe impact on global warming.^1^ Nearly quadruple the amount of investment, that is, $7.8 trillion, is projected for renewables over the next two decades compared with an estimated $2.1 trillion for fossil fuels to support rapid population, economic growth and global energy.^2^^,^^3^ Combined with the socioeconomic benefits, the solar energy industry has a vital role in the renewable energy revolution. Highly efficient organic-inorganic hybrid perovskite solar cells (PSCs) exhibit excellent photo-conversion properties, approaching the standard of widely used silicon-based cells.^4^^,^^5^ At the same time, the high power conversion efficiency (PCE) of PSCs, with their cost-effective materials and processes, makes them economically viable for commercialization. A single-junction PSC has reached 25.6% of its PCE at the laboratory scale, comparable to the first-generation monocrystalline silicon solar cell, which takes about 40 years for this level to achieve similar PCE. Besides, the perovskite-on-silicon tandem solar cell has achieved a PCE of 29.52%, with a device area of 30 cm × 30 cm. More recently, the all-perovskite tandem solar cell achieved a certified efficiency of 26.4%. The cell-to-module efficiency gap remains large, which could be the result of multiple factors.^6^ The non-uniformity of perovskite layers, including the pinholes and cracks, can lead to drops in the open-circuit voltage (V_OC_). The short circuit current (J_SC_) decrease is due to the unavoidable dead area and interconnections. Nevertheless, perovskite materials’ high moisture and UV light sensitivity impose a substantial challenge to their long-term PV performance. Despite this, the rapid progress in improving the PCE of organic-inorganic hybrid PSCs is probably unsurpassed in the history of photovoltaics (PVs), mainly due to optimizing the optoelectronic properties of the perovskite absorber itself.^7^^,^^8^

The trade-off between a PSC’s scalability and PCE remains challenging to balance, limiting further PSC commercialization. The levelized cost of electricity from PSCs is expected to decrease, making commercialization feasible shortly.^9^^,^^10^ However, although the efficiency and cost of PSCs are ideal for materialization, the lifetime of PSCs is yet to meet the requirements of a regular PV product. Moreover, almost all the high-efficiency PSCs are based on an active area of ≤0.1 cm^2^, and the scalability of the perovskite layer presents another risk of their large-scale fabrication.^11^

Yang et al. showed that as the cell dimension is increased from 0.12 to 1.1 cm^2^, the PCE of the PSC decreases from 17.5% to 15.5%.^12^ Recently, an unencapsulated TiO_*x*_N_*y*_-based cell exhibited a steady-state certified PCE of 22.82% after 250 h and under an inert atmosphere.^13^ Although the scalability and stability of high-performing PSCs are challenging, additional layers of protection are required to maintain their performance.^14^ An approach such as antioxidant additives and buffer layer incorporation mediates the crystallization rate, the design of perovskite cations, and the construction of low-dimensional perovskite structures to maintain the gradual PCE growth of a PSC, from 6% to 11%–13%, typically for small-scale cells.^15^^,^^16^^,^^17^^,^^18^

Despite being an excellent strategy to improve the performance of a PV device without compromising its cost, concentrated photovoltaic (CPV) technology with emplacement of parabolic mirrors or lenses usually serves as the focusing medium to boost the light intensity onto a smaller-area cell of the PV device, which generates high electrical power output. The optical arrangement also uses total internal reflection, which indirectly enhances the PCE.^19^ Besides, the Fresnel lens (FL) offers a tremendous level of device performance beyond the Shockley-Queisser limit of 30% PCE for a 1.6-eV single junction.^20^ PSC is envisioned to be a good choice for the FL. A recent report showed how four-junction III-V concentrator solar cells exhibited the highest PCE of 47.1% under the direct spectrum at 143 Suns.^21^ A conflicting challenge to deliver a highly cost-competitive CPV solution has also arisen. Owing to the continuing downward drive in conventional PV prices, III-V multi-junction CPVs are struggling to compete with the scale of growth occurring with flat-plate PV technologies. Moreover, the CPV technology allows fewer semiconductor components to achieve a high PCE, made from heavily mined and relatively rare metals.

Integrating the FL has tended to be thus far restricted to silicon and multi-junction solar cells. Hence, to contribute to a high PCE, PSCs need to be integrated with FL technology, which can be an alternative approach to addressing the scalability problem of PSCs and large-scale fabrication complications. However, perovskite stability is a pivotal factor interfering with their large-scale adaptation. Consequently, the popular perovskite compounds such as CH_3_NH_3_PbI_3_ (MAPbI_3_) and NH_2_CHNH_2_PbI_3_ (FAPbI_3_) fall short due to their thermal or structural instabilities. Therefore, mixing cations and halides has become an important design principle to achieve perovskite compounds with improved thermal and structural stability. As a solution, combining different cations can combine the advantages of the constituents while avoiding their drawbacks. A mixed cation perovskite, Cs_0.05_MA_0.05_FA_0.9_PbI_3,_ is a potential choice for use in FL applications instead of rapidly degraded MAPbI_3_.^22^ PSCs with mixed cations hinder the photo-bleaching issue of Pb-based perovskites, which results in enhanced device stability, retaining 95% of performance.^23^ Ding et al. reported one of the highest scalable performances of a planar PSC, exhibiting an average PCE of 15.63% with an area of ∼1.13 cm^2^.^24^

The FL is one of the typical primary concentrators because it is cost-effective and lightweight. However, most notably, its relatively high acceptance angle, optical efficiency, and high-temperature stability, compared with low or polymer-based concentrators, means that the FL is a facile option for integrating the PSCs at their “large-scale” level.^25^ Employing the FL can boost the PCE of PSCs from 21.1% under 1 Sun to 23.6% under 14 Suns irradiance, with a V_OC_ at 1.26 V, followed by a significant deterioration of the fill factor (FF).^20^ Alternatively, Baig et al. have achieved 21.6% under 1.78 Suns when coupled with low-concentrated optics, compared with 21% obtained under 1 Sun.^26^ Similarly, PSCs coupled with concentrated optics under 400 W/cm^2^ boosted the PCE to ∼90%, whereas under 1,000 W/cm^2^, PCE enhancement was ∼16% compared with PSCs without optics.^27^ A certified PCE of 22.6% for a PSC with an area of 1.02 cm^2^ was achieved by Jeon et al.^28^

In this study, a combination of experimental data and theoretical modeling analysis using COMSOL Multiphysics software was employed to understand the local temperature enhancement effect on the performance of the PSC module while it concentrated the sunlight. In addition, for modules of <200 cm^2^, a PCE of 18.0% with an area of 19.276 cm^2^ was certified in 2019 (Microquanta 2019).^29^ For 800- to 6,500-cm^2^ modules, a PCE of 17.9% with an area of 804 cm^2^ was reported in 2020.^30^^,^^31^ However, the advance over state-of-the-art technology for this work signifies a systematic PV performance evaluation with a high concentration ratio that permits maximum PCE enhancement up to a concentration factor while producing more power in innovative, cost-effective, and high-energy PV research into PSC. Integrating the FL resulted in stability in the output power of the cell’s static state; in contrast, the thermal effect of the cell under long-term dynamic conditions still poses a problem.^32^ Moreover, during the FL emplacement, the concentrated light raises the local temperature of the PSC and results in increased heat dissipation. To the best of the author’s knowledge, there has not yet been any experimental research on a “large-area” PSC to assess the variation of solar irradiance and its direct influence on the temperature of the FL-PSC system with an increase in the concentration ratio. The FL-PSC system systematically monitored a confluence of different incident illumination levels, geometrical concentrations, and thermal effects. It was found to generate a high electrical power output, conserving the system’s performance across different solar intensities and geometrical concentrations, allowing for an optimum scale-up without compromising the stability and cost of the device.

## Results and discussion

### Design and development of the “large-area” perovskite solar cell module for concentrated photovoltaic using the Fresnel lens

The illustration of the fabrication procedure, including all deposition processes and scribing steps of the PSC sub-cell, is illustrated in Figure S1.

**Figure 1:**
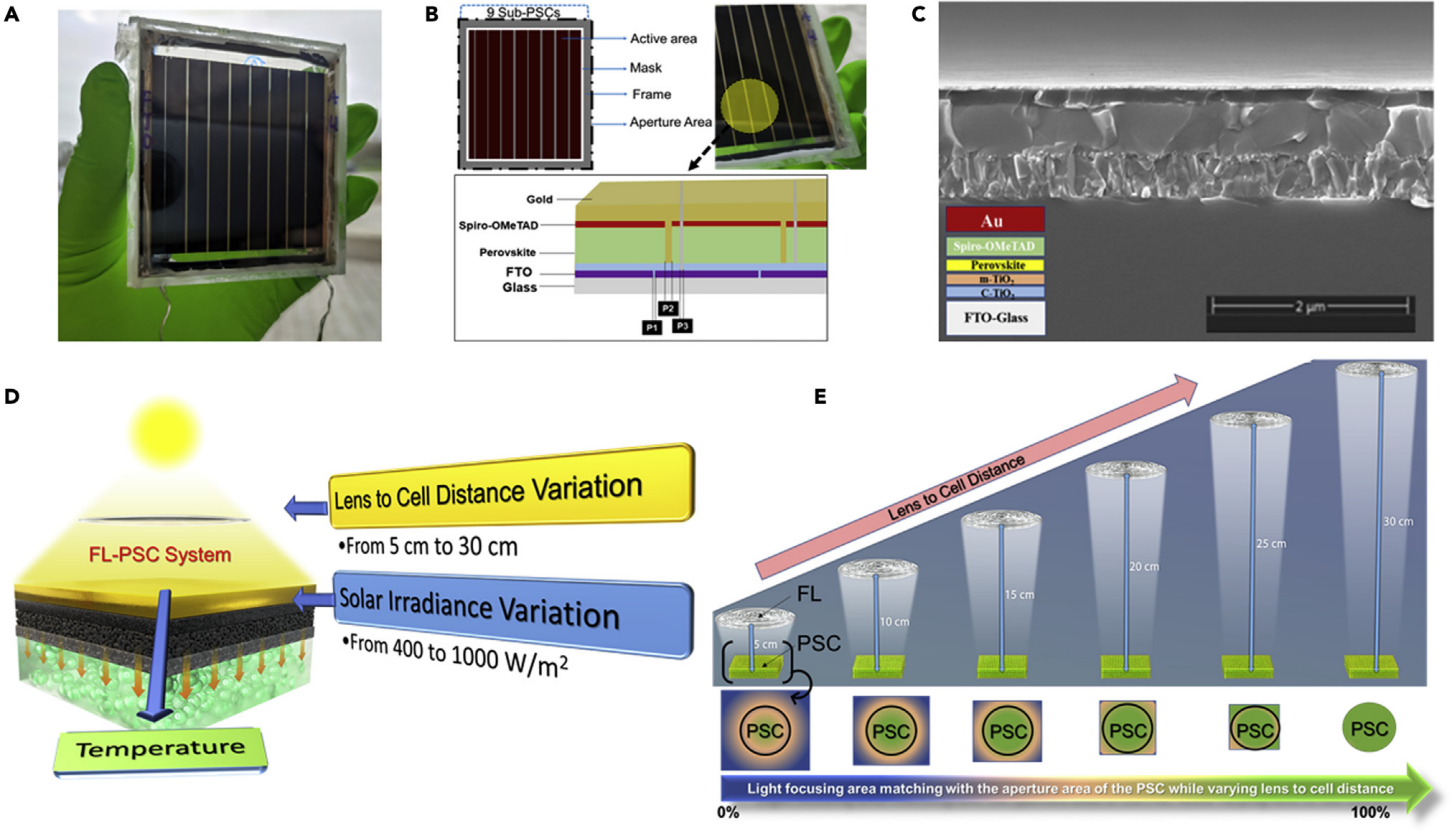
“Large-area” perovskite solar cell module development and understanding of the photovoltaic performance in concentrated sunlight PSC module development. (A–E) (A) A photograph of the encapsulated “large-area” of the PSC module; (B) a schematic representation of the module structure with nine sub-cells connected in series; (C) a cross-sectional SEM image of one of the sub-cells in the PSC module; (D) an operational testing view of the FL-PSC system, undertaken in this work; and (E) a schematic of the PV performance evaluation of FL-PSC system under various lens-to-cell distances capable of producing a focal spot relatively similar to the surface area of PSC, corresponding to their respective effective solar irradiance.

Figure 1A presents a photograph of the fabricated modules with nine sub-cells interconnected in a series, consisting of an aperture area of 28.56 cm^2^. A schematic illustration of the cross-sectional layer-by-layer device architecture corresponding to the planar n-i-p structure is shown in Figure 1B. Besides, the module’s cross-sectional scanning electron microscopic (SEM) image of the single-cell structure was also analyzed, as shown in Figure 1C. A superior engagement of associating layers can be clearly distinguished from the SEM cross-sectional image, resulting in the compact formation of the individual cells when fabricating the module. The concept of the FL-PSC system analysis is schematically shown in Figure 1D. The lens-to-cell distance variation of the FL and the solar irradiance variations were experimentally tested, followed by their direct effect on the temperature increase due to the concentrated sunlight. The distance between the FL and the PSC module varied from 5 to 30 cm at intervals of 5 cm (see Figure 1E). This FL is designed to operate optimally at a focal distance of ∼ 45 cm, producing a focal spot of around ∼ 2.8 cm in diameter.^33^ At the optimum focal length of ∼ 45 cm, the generated focal spot only covers 22% of the PSC, which causes a localized heat spot and non-uniformity distribution.^34^

Similarly, when the distance was <30 cm, the focal spot was beyond the module’s area. Consequently, at the focal length of 30 cm, the FL can produce a focal spot relatively like the aperture area of PSC through which the experimental setup was designed. As a result, distances of 10, 20, and 30 cm were selected to measure the FL-PSC system across a wide range of solar irradiance levels, from 400 to 1,000 W/m^2^, at an interval of 100 W/m^2^, respectively.

### Photovoltaic testing setup and conditions of the Fresnel lens-perovskite solar cell system

**Figure 2:**
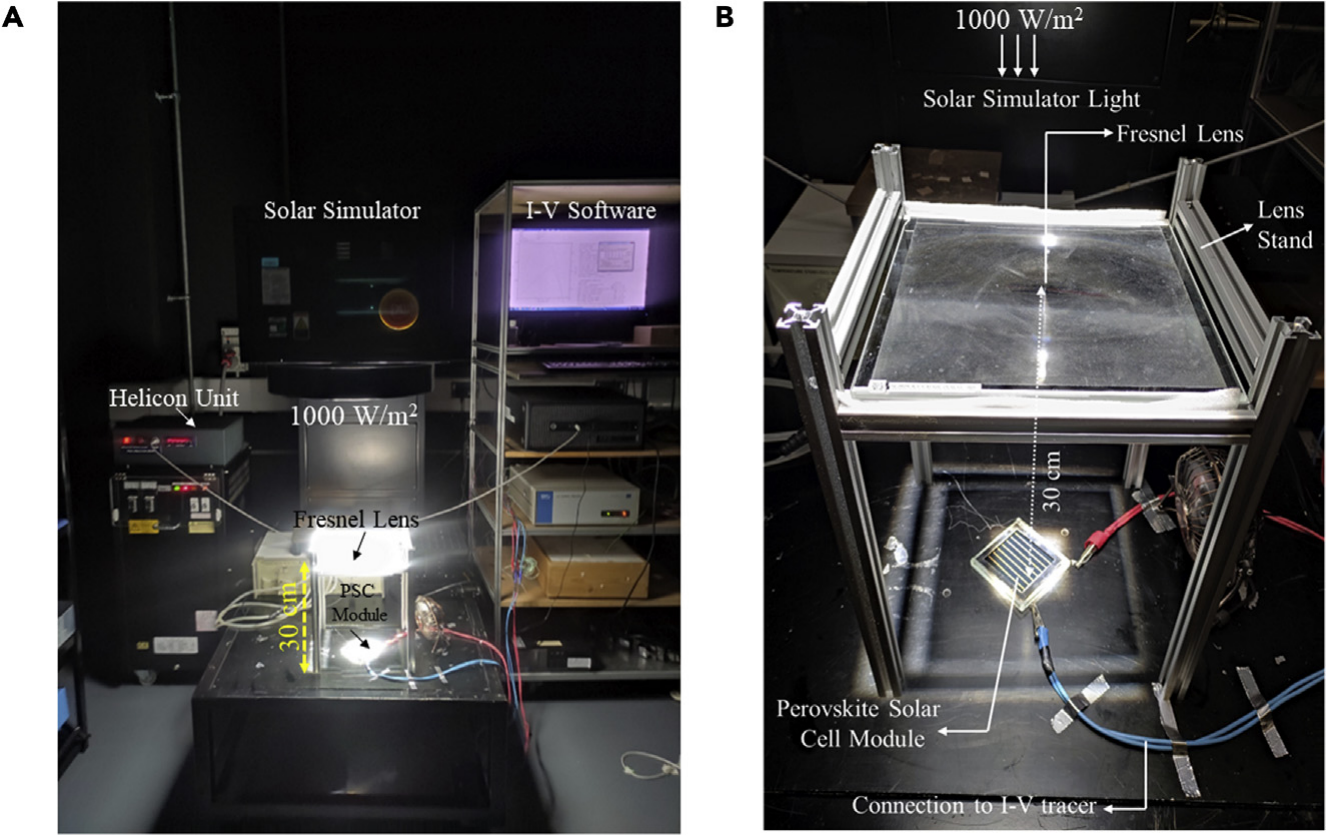
Experimental setup of Fresnel lens with the “large-area” PSC module tested under concentrated light using a solar simulator (A and B) The photographs are of the experimental setup of (A) the FL-PSC system and (B) the corresponding close-up view.

A silicon-on-glass refractive FL with an aperture surface area of 529 cm^2^ (23 cm × 23 cm) was fabricated by the ORAFOL and was introduced under an AAA continuous solar simulator manufactured by WACOM (Model no. WXS-210S-20), as shown in Figures 2A and 2B. The FL’s irradiance output is typically Gaussian contour distribution and can offer an optical concentration of up to 1,000 Suns without chromatic aberration. A detailed experimental setup is mentioned in the supplemental information. WACOM solar simulator consists of xenon short-arc lamp and two filters (UV and AM 1.5) to emulate a solar irradiance approximately to AM 1.5, as in Figure S2. The traced current and voltage are interpolated to determine the maximum power point and the FF. The instruments will record an error for any logged data out of these operating ranges, as mentioned in Figure S3. Noticeably, PV performance of the module is independent of its emplacement or orientation under the FL.

To attain different solar irradiances, a calibration plot was developed with the solar simulator, ensuring a strong linear correlation between the short circuit current (I_SC_) and the helicon value (solar irradiance) with the attenuation of the xenon lamp intensity as a function of the solar irradiance (see Figure S4). The equipment is calibrated with Fraunhofer-Institut für Solare Energie systeme, Freiburg, and developed according to the international standards of the world PV scale.^35^

Experimentally, the effective concentration ratio (*C*_*eff,exp*_) was characterized considering the measurements I_SC_ with and without the FL, as illustrated in Equation 1:

(Equation 1) $$C_{eff,\exp}=\frac{I_{SC,Concentrated}}{I_{SC,non-concentrated}}$$

The PCE of the FL-PSC system was calculated in Equation 2:

(Equation 2) $${PCE}_{\left(FL-PSC\right)=}\frac{P_{\max.}\;x\;100}{Aperture\;Area\;of\;the\;PSC\;mdoule\;x\;DNI\;x\;C_{eff,\exp}}$$

where P_max._ represents maximum power output (mW) and direct normal irradiance indicates the incident solar radiance (mW/cm^2^).

### Certified performance of the “large-area” PSC module

Five “large-area” PSC modules were fabricated to optimize PV performance. Figure S5 represents a comparative plot of the maximum PCE achieved for these modules. The champion module (see Figure 1A) is selected for further study. The laboratory-tested champion module was tested in the Fujian Metrology Institute Laboratory, China, for independent confirmation. The device showed a certified PCE of 19.92% during the forwarding scan (V_OC_ = 9.182 V, I_SC_ = 86.09 mA, and FF = 0.70) and 21.78% in the case of the reverse scan (V_OC_ = 9.065 V, I_SC_ = 86.09 mA, and FF = 0.78) of the PSC device, as represented in Figure S6.

### Photovoltaic performance of the concentrated perovskite solar cell module under various lens-to-cell distances

**Figure 3:**
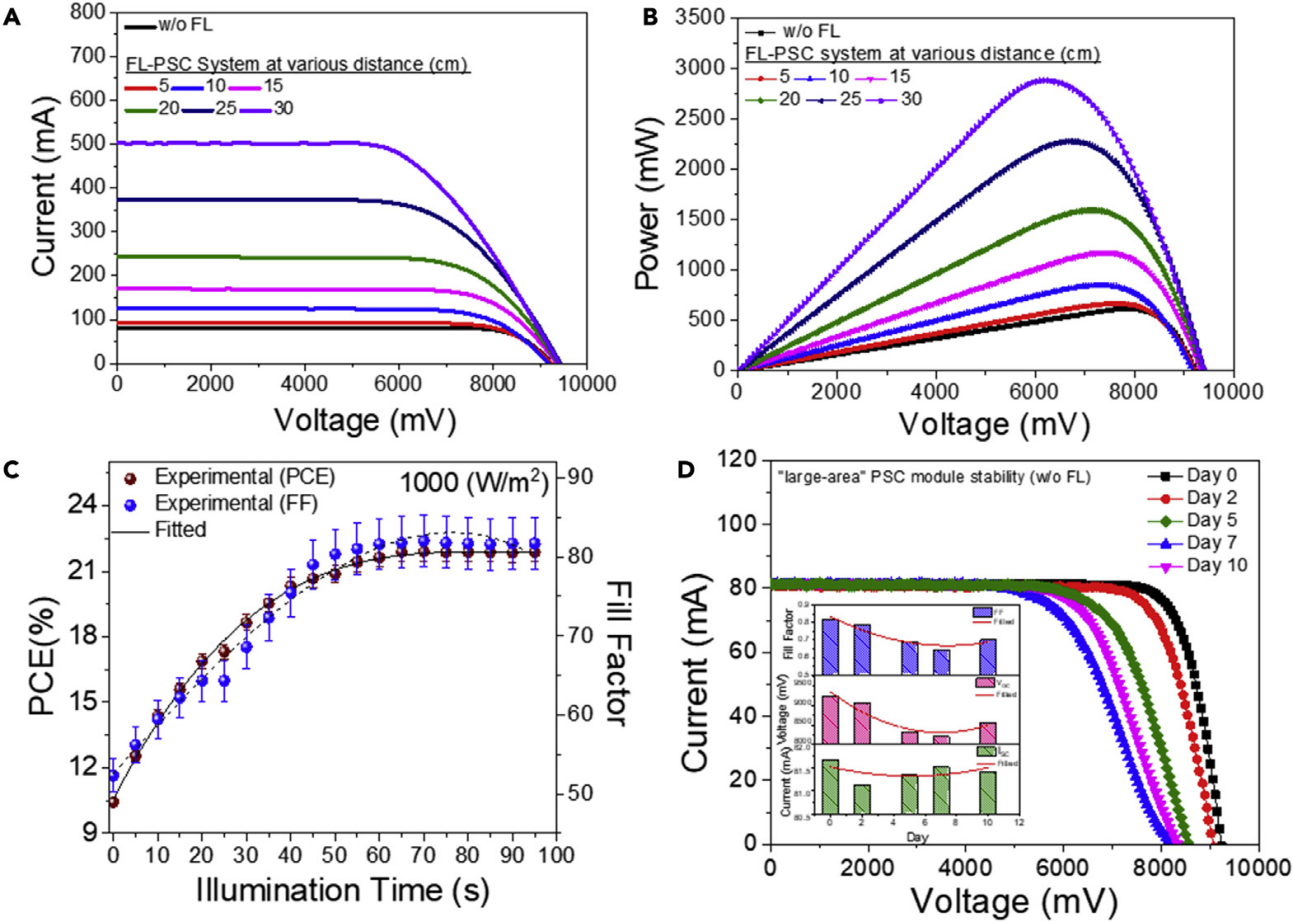
Effect of Fresnel lens emplacement on the perovskite solar cell module’s photovoltaic performance under 1 Sun condition while varying the lens-to-cell distance (A–D) (A) The current-voltage plot; (B) the corresponding power-voltage plot for the “large-area” FL-PSC system under different concentrated focal lengths under 1,000 W/m^2^ of solar irradiance, which is compared with the PSC-only module; (C) the light-soaking characteristics plot, where FF predominately governs the PCE; and (D) the day-wise current-voltage plot for stability monitoring under 1,000 W/m^2^ of solar irradiance of the PSC module (inset: corresponding PV parameter variation plot).

The champion PSC module exhibits a maximum PCE of 21.89% with I_SC_ of 81.67 mA, V_OC_ of 0.92V, and FF of 0.82 V. These PV parameters were optimized and checked repeatedly 20 times and were accordingly introduced with the FL to develop the FL-PSC configuration for further testing. Usually, the PSC absorbs a large photon spectrum under concentrated light that surges the photocurrent generation process, resulting in more significant PCE. During the experiment, a rapid improvement in I_SC_ and minor changes to the V_OC_ were observed after the FL emplacement. Figures 3A and 3B represent the current-voltage and corresponding power plot for the FL-PSC system during the lens-to-cell distance variation compared with the PSC module. The power output increases proportionally on the lens-to-cell distance resolution of the FL-PSC system. A steady power increment was observed of up to 8 cm of the focal length; subsequently, a massive power change was noticed and elevated to the maximum once the lens-to-cell distance reached 30 cm. The photovoltaic parameters are summarized in Table 1, where a maximum of 367% of power enhancement was noticed for the FL-PSC system. This significant power enhancement was dominated by the photocurrent increase of 515%, with a minor change to the V_OC_.

**Table 1:** Stabilized PV parameters of the Fresnel lens-integrated perovskite solar cell module for the different lens-to-cell distances at a fixed solar irradiance of 1,000 W/m^2^

Lens-to-cell distance (cm)	I_SC_ (mA)	I_SC_ variation (%)	C_eff_	V_OC_ (mV)	V_OC_ variation (%)	FF	FF variation (%)	PCE ±0.01 (%)	Power (mW)	Power variation (%)
0 (w/o Lens)	81.67	NA	NA	9,238	NA	0.82	NA	21.89	617.08	NA
5	93.25	+14.17	1.14	9,220	−0.2	0.78	−4.87	21.24	667.52	+8.17
10	125.05	+53.11	1.53	9,174	−0.7	0.74	−9.75	20.73	849.02	+37.62
15	170.09	+108.3	2.08	9,346	+1.16	0.74	−9.75	20.47	1,174.21	+90.28
20	242.53	+196.9	2.97	9,380	+1.53	0.70	−14.63	20.27	1,596.38	+158.69
25	373.64	+357.5	4.57	9,431	+2.09	0.64	−21.95	20.49	2,277.63	+269.09
30	502.78	+515.6	6.16	9,390	+1.65	0.61	−25.92	20.62	2,882.16	+367.05

NA, not applicable; w/o, without.

In contrast, a higher power of the system caused lower FF, showing a maximum of 26% loss during the lens-to-cell distance variation. One of the primary reasons for lowering the FF is temperature excitation due to concentrated ray traffic, but some loss may result from the photogeneration effect.^36^ However, the higher power may also cause leakage in the current due to carrier recombination in bulk at the interfaces of the PSC layers successively, which is increased during the variation of the lens-to-cell distances. High concentrated light caused the defect-induced recombination in the reduction of the FF and V_OC_, where the electron generates more while regenerating less.^37^ Figure 3C indicates the effect of incident light soaking, which significantly changes the FF and PCE of the PSC module under constant illumination as a function of time.^38^ The fabricated PSC module requires at least 50–60 s under constant illumination to achieve the best PCE of the device, with minimum sacrifice of its associated PV parameters, where FF is the significant one since it governs the stable PCE. Moreover, optimized light soaking is required to minimize the hysteretic behavior of the PSCs under constant illumination.^39^ As a result, the tested module is anticipated to exhibit negligible hysteretic behavior (see Figure S6, supplemental information) because of its light soaking time (50–60 s) and may also be due to its higher aperture area.

The stability of the PSC module was recorded for up to 10 days, as shown in Figure 3D. Factors including I_SC_ and FF exhibit meager changes, whereas ∼20% of V_OC_ loss was observed up to 10 days.

### Effect of solar irradiance on the photovoltaic performance of the concentrated perovskite solar cell module

**Figure 4:**
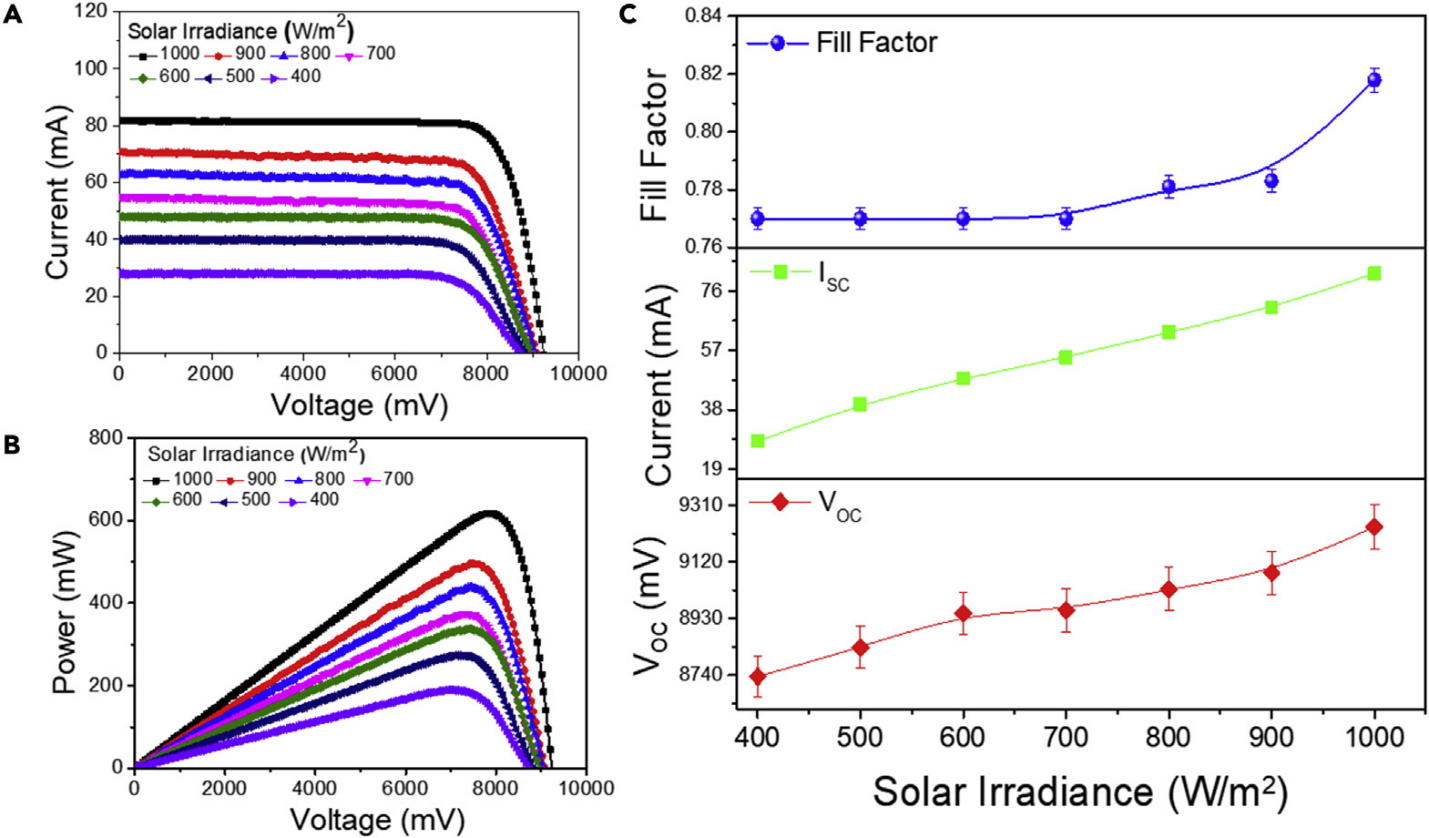
Effect of Fresnel lens emplacement on the perovskite solar cell module’s photovoltaic performance under different solar irradiances without Fresnel lens (A–C) Photovoltaic performance plots of (A) the current vs. voltage, (B) the power vs. voltage, and (C) the parameters’ trend bar plot for the I_SC_, V_OC_, and FF for the “large-area” PSC module under various solar irradiance levels.

The PV performance of the champion PSC module is further evaluated under various solar irradiance levels. The initial PCE of 21.86% was reduced by 16% as the solar irradiance was lowered from 1,000 to 400 W/m^2^. Alternatively, an improvement in the PCE of 37% and the power output of 163% is observed as the solar intensity is increased from 400 to 1,000 W m^−2^. Moreover, a sinusoidal characteristic was observed during the PCE measurement (from 21.8% to 16.6%). Figures 4A and 4B represent the current-voltage and corresponding power distribution performance plots.

In contrast, the module’s power exhibits a linear increment during the enhancement of solar irradiance from 400 to 1,000 W/m^2^, as shown in Figure 4C. Figure 4C represents a linear trend of I_SC_ improvement per the enhancement of solar irradiance, which offers a maximum enhancement of up to 70%. A negligible (5%) V_OC_ and FF alternation was observed during the enhancement of solar irradiance, as shown in Figure 4C. Interestingly the module power was periodically increased up to 70%, which is the case without the FL and only varying the solar irradiance.

Furthermore, the power enhancement was drastically boosted at the focal lengths of 10, 20, and 30 cm and thus was selected as a constant parameter during the variation of solar irradiation for each lens to cell distances. Therefore, the FL-PSC current-voltage system was tested under different solar concentrations to evaluate its PV performance across a wide range of solar irradiances. The following section discusses the PV performance and related parameters trend of the FL-PSC system. Table S1 represents a tabular form of all the PV measurements undertaken during the above mentioned conditions.

### Photovoltaic performance of the concentrated perovskite solar cell module under various solar irradiance levels at various lens-to-cell distances

**Figure 5:**
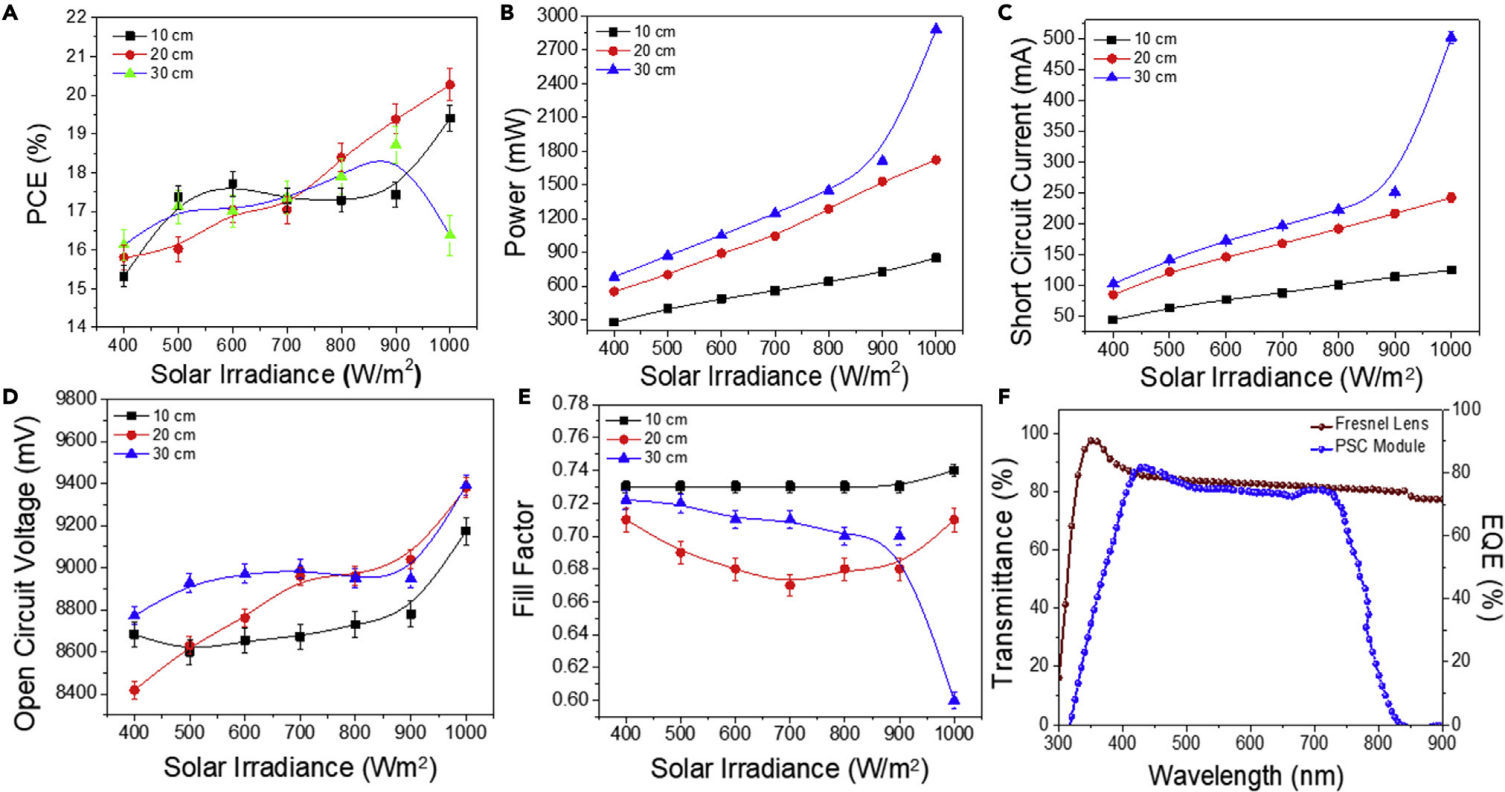
Effect of Fresnel lens emplacement on the “large-area” perovskite solar cell module’s photovoltaic performance under different effective solar irradiances at a lens-to-cell distance of 10, 20, and 30 cm (A–F) Photovoltaic performance plots of (A) power conversion efficiency, (B) power, (C) short-circuit current, (D) open-circuit voltage, and (E) fill factor as a function of solar irradiance for the Fresnel lens-perovskite solar cell system at a lens-to-cell distance of 10, 20, and 30 cm, and (F) the EQE of the perovskite solar cell module compared with the transmission spectrum of the Fresnel lens.

To further understand the effect of various solar irradiance levels at various lens-to-cell distances, the PV performance of the FL-PSC system was investigated. Figures 5A and 5B display the current-voltage and corresponding power distribution performance plots of the FL-PSC system during variations of solar irradiation at a lens-to-cell distance of 10, 20, and 30 cm with semilogarithmic trend. The PCE of the FL-PSC system was derived from Equation 2. Here, a maximum of 10% PCE enhancement (from 17.3% to 19.4%) was recorded during the solar irradiance changes from 400 to 1,000 W/m^2^; the module power was linearly increased by up to 67% from 278 to 849 W for 10 cm. These results indicate a massive power enhancement of the FL-PSC system without significant PCE deterioration. Similarly, FL integration dedicatedly uplifts the I_SC_ with a linear relationship with the additional solar irradiance (see Figure 5C). However, a moderate change in the V_OC_ (∼5%) was perceived (see Figure 5D), along with almost no change in the FF (see Figure 5E) during the enhancement of solar irradiation.

Furthermore, increasing the lens-to-cell distance to 20 cm signifies a direct linear relationship with the solar irradiance observed for I_SC_ and V_OC_ for the FL-PSC system (see Figures 5C and 5D). In contrast, the corresponding FF (see Figure 5E) exhibits a parabolic trend across the various solar irradiances. In addition, varying the solar irradiance from 400 to 1,000 W/m^2^ increased the PCE from 15.8% to 20.2%, with a 28% enhancement when the lens-to-cell distance was 20 cm. Besides, the module power was increased from 550 to 1,720 mW (see Figure 5B). This also signifies a linear relationship between I_SC_ and V_OC_ during the solar irradiation enhancement, offering a steady increment in the case of V_OC_, whereas a faster enhancement for I_SC_. However, the FF resulted in a minor parabolic change during the testing. Interestingly, the module power maintained its constant power enhancement of 69%, whereas the resultant power increased from 550 to 1,720 mW.

The PV performance was also evaluated in the case of 30 cm of the FL-PSC system. A steady sinusoidal trend was observed up to 900 W/m^2^ solar irradiances, followed by a massive power enhancement at 1,000 W/m^2^ (see Figure 5B). A high power generation was led by the I_SC_, which has increased ∼392% at 30 cm under 1,000 W/m^2^ solar irradiance, whereas the corresponding FF was dropped to 14% (see Figure 5E), followed by a slight (∼7%) enhancement of the V_OC_ (see Figure 5D). However, the overall effect of the PV parameters resulted in almost similar PCE (see Figure 5A). Noticeably, the power output again exhibits ∼76% increment, demonstrating a slightly higher growth similar to other distances (Table S1). A slight increment in the power of the PSC module at 30 cm is also due to maximization of the focal spot covering the area with the PSC module, which is relatively larger for <30 cm distances (see Figure 1E).

In this case, a similar trend for I_SC_ enhancement was also observed. However, the V_OC_ exhibited a parabolic trend, and the FF was slightly reduced while increasing solar irradiance. It is similar to the rate of electron-hole pairs, which effectively increase and vary linearly with solar irradiance. However, the capacity of the device for extracting the carriers to the external load is limited, probably due to limits in the conductivity of the hole transport or electron transport components of the device.

The PSC module shows ∼82% of the external quantum efficiency (EQE) at 300–1,000 nm as shown in Figure 5F. The transmittance spectrum of the FL strongly coheres with the EQE spectrum of the PSC model, as illustrated in Figure 5F. This signifies that the solar spectrum fits with the PSC module during this change of the concentrator. The FL transmits 90% of the light across 300–1,000 nm. The PSC module exhibits a broad visible EQE spectrum with an average value of 80%. The absorption of light <400 nm substantially reduces the system’s performance. Integrating the FL does not interfere with the PSCs.

### Photovoltaic parameters’ trend analysis, as observed during concentrated solar irradiance change for the “large-area” perovskite solar cell module

In general, the PCE of a solar cell increases when the solar irradiance decreases due to fewer carriers in the active layer and a lower recombination reaction. Interestingly, the PSC, as a single-junction solar cell, has shown a similar improvement in both PCE and power performance to the multi-junction solar cells. This evidence represents a potential for PSC to play an important role in CPV applications as an alternative for multi-junction SCs. The voltage and power relationship of the PSC module at different irradiance levels is as follows: as irradiance increases, the module can generate more power. The “large-area” PSC module significantly improves its power output under various solar irradiances. Interestingly, the power output increment was almost constant, irrespective of incident solar irradiance or concentrated focal lengths. Even the power output enhancement of the FL-PSC system sets a constant (68%) value, irrespective of its lens-to-cell distance.

This result signifies that the power output value can be increased while changing the solar irradiance and concentrated lens-to-cell distances using CPV; however, the maximum increment rate was almost constant under a specific concentrated lens-to-cell distance for various solar irradiance levels. As the concentration ratio of the system increases, it becomes increasingly difficult to maintain the uniformity of the incident flux on the solar cells.

The experimental relationship between the irradiance and the modules’ current and power can be expressed as in Equation 3:

(Equation 3) $$\frac{I_{1}}{I_{2}}≅\frac{I_{SC1}}{I_{SC2}}≅\frac{P_{1}}{P_{2}}$$

where I indicates incident solar irradiation, I_SC_ and P represent the devices’ short-circuit current and corresponding power output, and 1 and 2 indicate the two consecutive measurements.

Alteration of V_OC_ is observed throughout the experiment, possibly due to the heat generated by the internal power dissipation during energy production. V_OC_ secured an average minimum change of 5% and a maximum of 10%. However, the decline of the V_OC_ value was maximized when the concentrated lens-to-cell distance reached 20 cm. The V_OC_ exhibited a minor logarithmical increment with light intensity (∼2%) when the concentrated lens-to-cell distance was 30 cm. In addition, a reduced charge density at the interfaces suppresses interfacial recombination and yields higher V_OC_ and FF. These factors combine to yield significantly enhanced PCE at higher solar irradiance levels and follow the trend under concentrated solar irradiance. Another factor may be the light-soaking characteristics of the PSC module. The light-induced phase segregation that typically occurs for PSC enhances the carrier extraction ability and may be promoted at higher solar irradiance levels. FF deficiency (with a maximum of 2.8%) was further observed under concentrated sunlight due to the enhanced carrier recombination in the interface region, leading to high carrier density of PSC layers and further series resistance.

The relevant PV performance of the FL-PSC system signifies the device’s photocurrent proportionally with the solar concentration and hence solar flux, whereas the photovoltage scales logarithmically with the flux concentration depending on lens-to-cell distance. However, there are increases at high flux concentrations if the spurious heating and series resistance losses are appropriately diminished. These observations demonstrate the potential of flux concentration, which is highest at 30 cm distance, to enhance the photocurrent and/or reduce the applied bias in the FL-PSC system.

As the PSC has several defects that can affect charge transport and recombination of photo-generated carriers at higher solar irradiance levels and thus concentrated light, these defect states can be minimized to generate better performance.^40^^,^^41^ Ion migration is also a general PSC phenomenon with significant performance impacts. For example, the Li^+^ ion, used with the spiro-OMeTAD, contained a high diffusion tendency, even faster than with the perovskite ions at higher solar irradiance levels; therefore, an accumulation of Li^+^ in TiO_2_ facilitates electron injection in the electron transport layer (ETL) and thus improved efficiency.^42^ It is anticipated that the variation observed for various solar irradiance levels is due to a marginal difference in the band gap of the PSC materials, with the light intensity significantly influencing its power generation. Moreover, an enhanced charge density in the low-light region corresponds to a constant capacitance, indicative of a charge accumulation on device contacts. Thus, the power output of the PSC module increases linearly with the device I_SC_. Besides, the concentrated light undergoes heating on the device, resulting in rapid temperature enhancement, affecting the PV performance of the PSC.^43^^,^^44^

In our previous study with the low concentrated PSC at different solar irradiance levels, it was observed that there was a PCE degradation phenomenon under the high concentration assisted by electron injection into the ETL, trapped by unoccupied sides. The high diffusion length and lower carrier recombination characteristics of the perovskite enable a fit that can be used as an absorber in CPV. However, V_OC_ reached its saturated value with increasing light intensity and lens-to-cell distance. The ETL-perovskite interface degrades or generates trap density (monomolecular recombination rate) when exposed to high incident light or low open-circuit conditions. This is due to less sunlight, thus losing its expected PCE outcome. This is because its charge-carrier migration becomes steady, and hence degradation may be restricted.

In contrast, the device can avoid the high-temperature generation of concentrated light, which may provide more thermal stability. Therefore, this photo-physical deportment synergistic effect resulted in a PV performance variation of the FL-PSC system at different solar irradiance levels. Moreover, FL produces considerable spherical and chromatic aberrations that lead to a non-uniform spot. The characteristics of the non-uniform spot vary as a function of the distance from the lens.

Recent achievements of CPV-PSC combined systems have been highlighted in Table 2, indicating that CPV integration enables a great scope to minimize the device area-performance trade-offs for PSCs. However, most studies are limited to smaller area-based PSC devices and low concentrated-based PVs. In contrast, this work signifies a preliminary investigation into “large-area” PSCs, which can be adopted for the FL. The FL-PSC system results for this work show a scope of PSC scalability due to it excellent PV performance under a simplified integration with cost-effective parabolic lenses such as the FL in this case.

**Table 2:** Comparison of reported achievements for concentrated PSCs

Entry No.	Major PSC architecture	Area	CPV	Achievement	Reference
1	SnO_2_/PC_61_BM/FA_0.79_MA_0.16_Cs_0.05_PbI_2_.7Br_0.3_/spiro-OMeTAD-PTAA/Au	0.87 cm^2^	Neutral density filters	• 23.6% PCE under 14 Suns when compared with 21.1% PCE under 1 Sun, and the measured open-circuit voltage of 1.26 V under 53 Suns	Wang et al.^20^
2	SnO_2_/PC_60_BM/Cs_0.15_FA_0.85_PbI_3_/spiro-OMeTAD/Au	3.2 mm^2^	High-concentrator (under the glove box)	• The first step for perovskites in CPV realization • 18% PCE at 1 Sun and 16% PCE at 13 Suns • A minor effect of light soaking	Troughton et al.^22^
3	TiO_2_/SnO_2_-(FAPbI_3_)0.875(MAPbBr_3_)0.125(CsPbI3)0.1) by mixing lead iodide-spiro-OMeTAD-Au	9 mm^2^	Low-concentrator	• 21.6% of PCE under 1.78 Suns • Series resistance enhancement at higher irradiance • Working effectively under a lower DNI and diffuse radiation	Khalid et al.^26^
4	SnO_2_/PC_61_BM/FA_0.79_MA_0.16_Cs_0.05_PbI_2.7_Br_0.3_/spiro-OMeTAD-PTAA/Au	–	Parabolic mirror	• High V_OC_ up to 1.4 V under concentrated light • Suppression of trap-mediated (Shockley-Read-Hall) recombination of charge carriers as the key exceeds the Shockley-Queisser limit	Lin et al.^45^
5	TiO_2_/Cs_0.05_(FA_0.9_MA_0.1_)_0.95_Pb(I_0.9_Br_0.1_)_3_/spiro-OMeTAD-Au	6 mm^2^	Fresnel lens	• Thermoelectric generator • 23% power conversion efficiency under 10 Suns	Zhou et al.^46^
6	TiO_2_/Cs_0.05_MA_0.05_FA_0.9_PbI_3_/spiro-OMeTAD-Au	28.58 cm^2^	Fresnel lens	• Certified high PCE (21%) for “large-area” PSC module • Maximum power output achieved at 2,880 mW. The Fresnel lens integration resulted in a constant power generation rate for different solar irradiance levels at various lens-to cell-distances	Current Work

DNI, direct normal irradiance; PC_61_BM: 4-(1,3-dimethyl-2,3-dihydro-1H-benzimidazol-2-yl)-*N*,*N*-diphenylaniline (*N*-DPBI)-doped phenyl-C_61_-butyric acid methyl ester; PTAA, poly[bis(4-phenyl)(2,5,6-trimethylphenyl)amine]; spiro-OMeTAD, 2,20,7,70-tetrakis[*N,N*-di(4-methoxyphenyl)amino]-9,90-spirobifluorene.

### Experimental thermal analysis for the concentrated perovskite solar cell module

**Figure 6:**
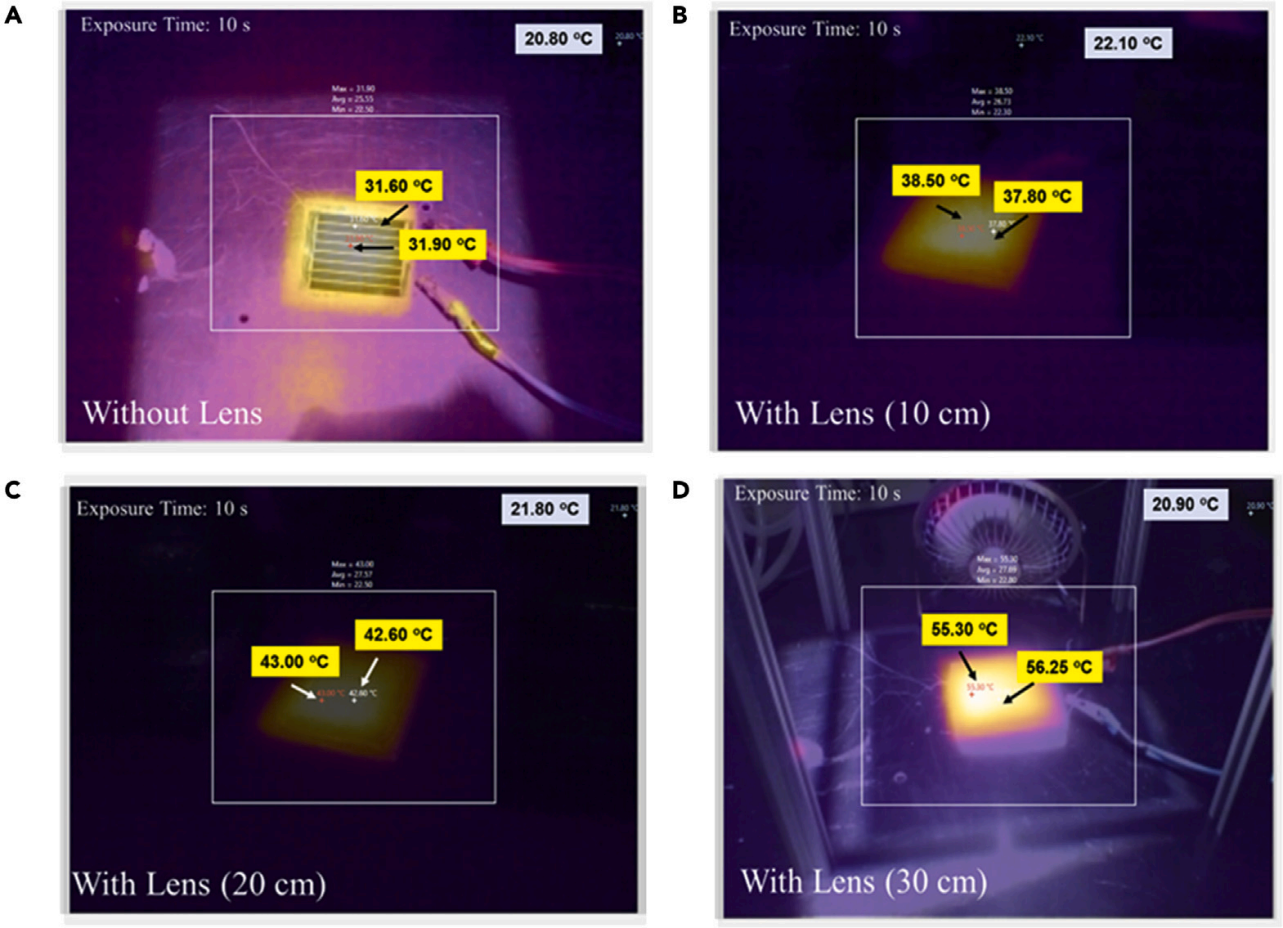
The surface temperature detected during the Fresnel lens-induced concentrated light on the “large-area” perovskite solar cell module (A–D) Infrared thermal images of the “large-area” concentrated perovskite solar cell module: (A) the PSC module only; the Fresnel lens-PSC module at a lens-to-cell distance of (B) 10 cm, (C) 20 cm, and (D) 30 cm, under 1,000 W/m^2^ of solar irradiance.

One of the main challenges in the CPV system is keeping cell temperatures within the average operating temperature. It should be noted that light absorption inevitably causes heat generation and temperature increases. Therefore, an increase in the light intensity also increases the heat load on the cell, which may be detrimental to cell performance. Predicting the temperature of PSC in CPV system is crucial for performance analysis and characterization. Increasing the concentration ratio elevates the temperature of the cell. Thermal infra-red images of the FL-PSC system were captured under different conditions. The surface temperature of the FL-PSC module increased from 21°C to 31°C following a 10-s irradiation of 1,000 W/m^2^ (see Figure 6A). A rapid increase in the surface temperature was also observed under concentrated light for 20- and 30-cm conditions under 1,000 W/m^2^ irradiation (see Figures 6B and 6C). The surface temperature reached 56°C at the lens-to-cell distance of 30 cm (see Figure 6D). Therefore, the encapsulated PSC module restricts the perovskite films’ degradation, offering a superior power output at a steady-state temperature of 88 °C.

### Thermal modeling of the concentrated perovskite solar cell module

PSCs exhibit maximum PCE around room temperature, with their performance impaired at higher and lower temperatures. However, temperature is a crucial external factor that dominates the charge mobilization across the interface of the individual layers and results in the accumulation of ions at selective interfacial contacts during temperature stress.^43^

Considering the effect of temperature originating from the concentrated light, the PV performance of the module was modeled using COMSOL Multiphysics software-based thermal transient analysis.^33^ The FL, which concentrates light into a focal point, thus raises the temperature predominately for the focal point. As a result, the concerning thermal modeling depicts attempted heat dissipation across the PSC surface according to a particular time.

**Figure 7:**
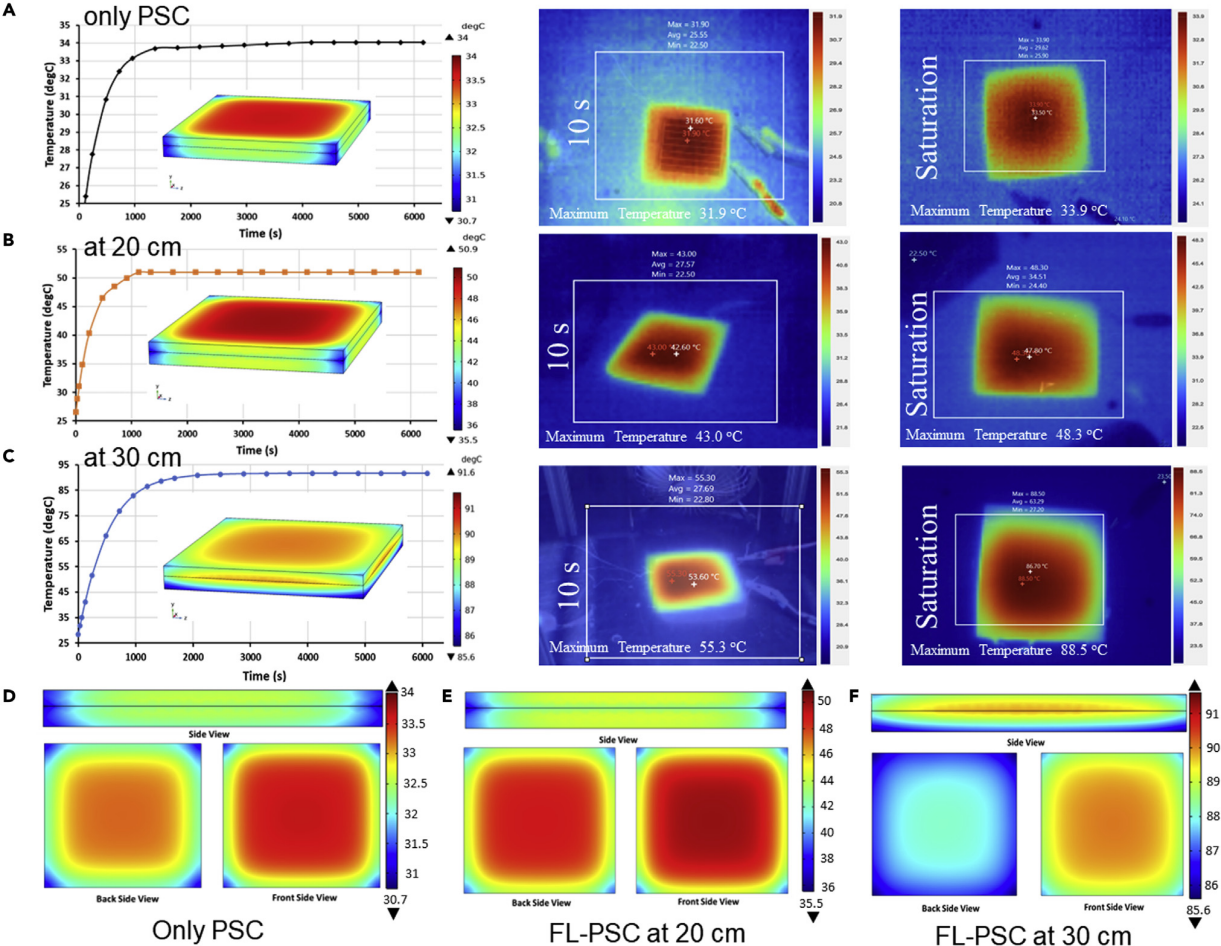
Temperature and heat-transfer modeling analysis across the perovskite solar cell interfaces during a lens-to-cell distance at an incident solar irradiance of 1,000 W/m^2^ The COMSOL simulates the thermal profile of the FL-PSC system under 1,000 W/m^2^. Thermo-kinetic COMSOL simulation results of the “large-area” PSC module for (A) without the FL and with the FL-PSC system, with the FL-PSC system at a lens-to-cell distance of (B) 20 cm and (C) 30 cm; and interfacial temperature analysis of the “large-area” PSC module for (D) without the FL and with the FL-PSC system at a lens-to-cell distance of either (E) 20 cm or (F) 30 cm.

The simulation was a prior process to indoor physical experimentation. The reason for that was the instability of PSC under high-temperature levels resulting from the concentration of the FL. Therefore, the COMSOL model results estimated the expected maximum temperature for the PSC to ensure before experimenting that the level of temperature is within the accepted range or that a cooling arrangement mechanism is in need. The thermo-kinetic PV performance of the “large-area” module is shown in Figure 7. The PSC module’s boundary conditions and parametric assumptions are given in Figure S7. A steady surface temperature upgradation reached a maximum of 33.5°C under 1,000 W/m^2^ (see Figure 7A). The FL-PSC module’s surface temperature became saturated for up to 6,000 s. Corresponding experimental surface thermal images (captured after their saturated temperature was reached) corroborate the simulations. At the 20 and 30 cm lens distances, concentrated conditions genuinely passivate the temperature, as shown in Figures 7B and 7C. Within 1,000 s, the surface temperature of the module reaches a maximum value of 50°C for 20 cm. While, at a distance of 30-cm, the surface temperature reaches 87°C. During the measurements, perovskite layer degradation was a significant concern.

Figure 7D shows the cross-sectional thermal analysis of the PSC module, where the temperature remained in a lower range of 30°C–35°C. Under concentrated conditions, the interface temperature was stretched from 35°C to >50°C (for 20 cm, see Figure 7E), and a higher-temperature small window of 35°C to >91°C was predicted (for 30 cm; see Figure 7F). Until now, the high-temperature application of PV technologies is primarily found in the space environment for conventional cells, which are relatively expensive, and complex compared with PSC.^47^ The FL-PSC system could be explored for the high-temperature region as the current modeling exhibits a saturated temperature for concentrated light that can reach a maximum of 88°C, maintaining a high-power output. The simulated maximum temperature in COMSOL and the measured maximum temperature showed excellent comparability with a discrepancy of 0.3%, 5.1%, and 3.4% for PSC with no concentration, 20, and 30 cm lens-to-cell distance, respectively. Perovskite layer may undergo degradation at > 60°C, indicating structural stability may not be the prime factor in determining device stability.^48^

### Conclusion

“Large-scale” PSC modules were developed, and the champion module exhibits a maximum PCE of 21.89%, whereas the same module received a 19.9% certified PCE from an independent international testing source. Hence, the high PCE of a PSC when using the FL technology for higher PCE generation of an SC leads the way toward novel interdisciplinary PV research. Furthermore, it has been experimentally demonstrated that integrating the FL as a CPV with the perovskite-based “large-area” modules exhibits a high-power output for the first time. The FL-PSC performs well under various concentrated focal points, followed by different solar irradiance levels, by sacrificing a minor FF and an open-circuit voltage while increasing the short-circuit current and power output. Besides, the FL-PSC system resulted in a unique observation with its high-power output, and the rate of power enhancement was secured at a nearly constant trend, similar to the results of not having the FL. Despite increasing the device’s area by > 500 times, the almost insignificant loss has been observed under various concentrated light conditions. The recorded concentrated solar power of 2,880 mW under 1,000 W/m^2^ at a 30 cm lens-to-cell distance thus envisages its potential marketization. Using the FL for PSC, the effect of temperature across the PSC layers is also validated using the COMSOL simulation approach. It is anticipated that PV performance of the FL-PSC system is influenced by external (solar irradiance levels and temperatures) and internal factors (ion migration, light-soaking, and carrier recombination).

With the PV industry gaining impetus in power production recently, several new companies are now coming forward to introduce the straightforward CPV technology with the high-efficiency PSC system, which can effectively produce electricity and readily compete with conventional electricity costs. However, this is understandable as the technology is relatively new, and the conventional FL and parabolic concentrators are the most tested and proven in this domain; hence, the FL-PSC system lends itself to be studied in further detail. Understanding the initial experimental assessment of the FL-PSC enables suitable design and thermal management strategies for the light-concentrating effects at the perovskite interfacial, which requires further understanding of its long-term operational stability under high levels of irradiance and elevated temperatures. In addition, this study was designed to consider only a refractive optic and bare PSC without incorporating secondary optics, such as a homogenizer. The effect of non-uniformity and secondary optics might be researched further in another study.

## STAR★Methods

### Key resources table

**Table:** 

REAGENT or RESOURCE	SOURCE	IDENTIFIER
**Chemicals, peptides and recombinant proteins**		

FTO glass	Asahi, 12–13 Ω/cm^2^	https://www.agc.com/en/products/electoric/index.html
Titanium diisopropoxide bis(acetylacetonate)	Sigma Aldrich	CAS Number: 17927-72-9
dimethylformamide (DMF)	Sigma Aldrich	CAS Number: 4637-24-5
dimethyl sulfoxide (DMSO)	Sigma Aldrich	CAS Number: 67-68-5
bis(trifluoromethane)sulfonimide lithium salt	Sigma Aldrich	CAS Number: 90076-65-6
Cobalt-complex (FK209)	Sigma Aldrich	Product Number: 805394, CAS number: CAS:1447938-61-5
Methylammonium iodide (MAI)	GreatCell Solar	SKU MS101000, CAS Number: 14965-49-2
formamidinium iodide (FAI)	GreatCell Solar	SKU MS150000, CAS Number: 879643-71-7
methylammonium chloride (MACl)	GreatCell Solar	SKU MS601000, CAS Number: 593-51-1
phenethylammonium iodide (PEAI)	GreatCell Solar	SKU MS109200, CAS Number: 151059-43-7
TiO_2_ paste (Dyesol-30NR-D)	GreatCell Solar	SKU MS002300
Spiro-OMeTAD, (N^2^,N^2^,N^2^′,N^2^′,N^7^,N^7^,N^7^′,N^7^′-octakis(4-methoxyphenyl)-9,9′-spirobi[9H-fluorene]-2,2′,7,7′-tetramine	Xi’an Polymer Light Technology in China	CAS Number: 207739-72-8
CsCl	Xi’an Polymer Light Technology in China	CAS Number: 7647-17-8
PTAA	Xi’an Polymer Light Technology in China	CAS Number: 1333317-99-9
PbI_2_	TCI Europe	CAS Number: 10101-63-0

**Deposited data**		

Laser Patterned	Speedmarker 300, Trotec	https://www.troteclaser.com/en-gb/laser-machines/laser-marking-machine-speedmarker
Solar Simulator	WACOM AAA [Model no. WXS-210S-20	https://www.helmholtz-berlin.de/projects/pvcomb/analytik/sosi/index_en.html
SEM	FEI Sirion-200	https://caeonline.com/buy/scanning-electron-microscopes/philips-fei-sirion-200/9267884
I-V curve tracer	EKO: MP-160	https://www.eko-instruments.com/eu/categories/products/iv-measurement-instruments/mp-160-i-v-tracer
IPCE	BENTHAM PVE300	https://www.bentham.co.uk/products/systems/pve300-photovoltaic-eqe-ipce-and-iqe-solution-16/
Fresnel lens	Orafol	https://www.orafol.com/en/europe/products/optic-solutions/productlines
Thermal images	FLIR T425	http://www.merlinlazer.com/T425-Thermal-Imaging-Camera

**Software and algorithms**		

Graph Plotting	Origin Lab	https://www.originlab.com/index.aspx?go=PRODUCTS/Origin
Thermal Modeling	COMSOL Multiphysics Software	https://www.comsol.com/comsol-multiphysics

### Resource availability

#### Lead contact

Further information and requests for resources and reagents should be directed to and will be fulfilled by the lead contact, Tapas K. Mallick (t.k.mallick@exeter.ac.uk).

#### Materials availability

This study did not generate new materials. All chemicals were obtained from commercial resources and used as received.

### Experimental model and subject details

This work did not need any unique experimental model.

### Method details

#### Fabrication of “large-area” perovskite solar module

The triple cation, Cs_0.05_MA_0.05_FA_0.9_PbI_3_, perovskite was used to fabricate the individual PSC employing mesoporous TiO_2_ as an electron transport layer (ETL) and Spiro-OMeTAD, (N^2^,N^2^,N^2^′,N^2^′,N^7^,N^7^,N^7^′,N^7^′-octakis(4-methoxyphenyl)-9,9′-spirobi[9H-fluorene]-2,2′,7,7′-tetramine, as a hole transport layer, and with Au as a back electrode, as reported previously.^48^ The illustration of the fabrication procedure, including all deposition processes and scribing steps of the PSC sub-cell, is illustrated in Figure S1, SI. In detail, fluorine-doped tin oxide (FTO) substrate (Asahi, 12–13 Ω/cm^2^) with a size of 65 mm × 70 mm^2^ was patterned with laser (Speedmarker 300, Trotec) for P1. The patterned FTO substrate was sequentially cleaned with detergent, deionised water, acetone, and isopropanol in the ultrasonic bath for 15 min, respectively. The FTO substrate was cleaned further with Ultraviolet-Ozone surface treatment for 15 min. A compact TiO_2_ layer (c-TiO_2_) was deposited on the clean FTO substrate by spray pyrolysis with a precursor solution (1.0 mL of Titanium diisopropoxide bis(acetylacetonate) in 24.0 mL of isopropanol) at 450 °C. The substrate was then annealed at 450 °C for 30 min. After the substrate cooled down to room temperature, a mesoporous TiO_2_ layer (*m*-TiO_2_) was deposited by spin-coating a TiO_2_ nanoparticle paste diluted in anhydrous ethanol at a weight ratio of 1:12 at 3,000 rpm for 20 s. The substrate was then heated at 125 °C for 10 min and sequentially sintered at 500 °C for 30 min. A triple cation perovskite, Cs_0.05_MA_0.05_FA_0.9_PbI_3_, precursor solution (1.4 M) was prepared by adding 645.4 mg of PbI_2_, 216.7 mg of formamidium iodide (FAI), 11.1 mg of ethylammonium iodide (MAI), and 11.8 mg of CsCl to 200 μL of N, N′-dimethylsulfoxide (DMSO), containing 800 μL of dimethyformamide (DMF). Before each step, a UV-ozone treatment of the substrates was applied for 15 min at room temperature. Afterward, in a two-step process, 40 μL of perovskite precursor solution was spin-coated on top of the c-TiO_2_/m-TiO_2_ substrate. The first step was 1,000 rpm for 5 s with an acceleration of 200 rpm/s. The second step was 4,000 rpm for 20 s with a ramp-up of 1,000 rpm/s. The as-obtained liquid perovskite film was placed in purpose-built rapid vacuum drying equipment. After pumping for 20 s, a brown, somewhat transparent perovskite film with a mirror-like surface was obtained. The fresh perovskite layer was annealed at 100 °C for 1 h and then at 150 °C for 10 min. After cooling to room temperature, 100 μL of phenethylammonium iodide (PEAI) solution (5 mg/mL in isopropanol) was dropped on the spinning perovskite layer at 5,000 rpm for 20 s. Then, 300 μL of Spiro-OMeTAD, Spiro-OMeTAD) solution, dissolving 100 mg of Spiro-OMeTAD with Li-TFSI, Lithium bis(trifluoromethanesulfonyl)imide (23 μL from a 520 mg mL^−1^ stock solution in acetonitrile), TBP, tributyl phosphate (39.0 μL), and Co(II)TFSI, (Tris(2-(1H-pyrazole-1-yl)pyridine)cobalt(II) Di[bis(trifluoromethane)sulfonimide) (18 μL from a 376 mg mL^−1^ stock solution in acetonitrile) as dopants into 1.279 mL of CBZ (benzyl chloroformate) was spin-coated onto the perovskite layer at 3,000 rpm for 20 s. The P2 lines (a width of 600 μm) were patterned before the Au evaporation process step, with an average laser power of 15% under a speed of 1,000 mm/s and a frequency of 65 kHz for a pulse duration of 120 ns. The distance between P1 and P2 was about 100 μm. When a 70 nm-thick Au layer was deposited, the P3 line (a width of 200 μm) was fabricated under the same scribing condition as the P2 line. The distance between P2 and P3 was also about 100 μm. Since the PSC performance causes an effect at high temperatures, a pressurised air blow fan optimised system was used to reduce the temperature at higher concentration to minimise any thermal stress to the device and to avoid any unwanted effects. The thickness of the c-TiO_2_/m-TiO_2_ layers is about 100 nm, capped with about 700 nm of perovskite film and 230 nm of Spiro-OMeTAD as a hole transporting layer of 70 nm of Au as an electrode (Figure 1C).

#### Solar simulator set up and Fresnel lens characteristics

The PSC module directly interacts with a stainless-steel sheet coated with black (the setup base of the WACOM Solar simulator by the manufacturer) to absorb the most thermal energy and reflect the minimal visible light radiation. The utilised solar simulator is manufactured by WACOM [Model no. WXS-210S-20]. WACOM solar simulator consists of Xenon short-arc lamp and two filters (UV and AM 1.5) to emulate a solar irradiance approximately to AM 1.5. This solar simulator is categorised as class AAA and simulates solar intensity within ±2% non-uniformity range. Before experimentation, the solar simulator was switched on for 15 min to warm up. Then, the calibration must be conducted relying on either the pyranometer solar sensor or the solar cell provided by the solar simulator manufacturer to attenuate the intensity of the solar simulator Xenon short-arc lamp to generate a solar irradiance of 1000 W/m^2^. Before experimentation, the solar simulator was switched on for 15 min to warm up. Then, the calibration must be conducted relying on either the Pyranometer solar sensor or the solar cell provided by the solar simulator manufacturer to attenuate the intensity of the solar simulator Xenon short-arc lamp to generate a solar irradiance of 1000 W/m^2^. The I-V curve tracer EKO: MP-160 instrument was utilised to perform the electrical measurements based on the open-circuit condition technique. The simulated solar irradiance of 1000 W/m^2^ on the PSC can be measured simultaneously as both current and voltage products instantaneously over a dedicated time interval. The traced current and voltage are interpolated to determine the maximum power point (MPP) and the fill factor (FF). The I-V curve tracer EKO: MP-160 instrument operates in a current range between 0.005 A and 10 A and a voltage range between 0.5 V and 300 V. The temperature increase is in proportional correlation with a growth in the focal distance up to ∼30cm as many solar rays are concentrated into the PSC rather than being lost. In addition, the electrical metallisation of PSC has its limit to the flow of electric current in correlation with concentrated rays; thus, the series resistance of PSC might be an additive factor to the heat built up on the cell. A Fresnel lens’s irradiance output is typically Gaussian contour distribution and can offer an optical concentration of up to 1000 suns without chromatic aberration. However, this Fresnel lens has excellent uniformed output across at least 1 cm in the center at the optimum focal length. Therefore, the PSC has been positioned in the center of the generated focal spot. The resultant optimum focal spot and focal distance from the Fresnel lens are ∼2.8 cm and ∼45 cm, which was thoroughly investigated across its contour surface area thermally and electrically and showed a relatively excellent performance across the focal spot diagonal (see Figure 1E).

A calibration plot (Figure S4) of the solar simulator was used to ensure the strong linear correlation between the ISC and the helicon value with the attenuation of the Xenon lamp intensity and the consistency of the solar irradiance. The irradiance changes with a complicated and controlled circuit, an integral part of the WACOM simulator, regulated by a combination of voltage and resistance changes; each irradiance profile is calibrated according to the base results. Any irradiance profile changes don’t impact the spectrum, uniformity, or collimation; all of these categories are within AAA quality.

### Quantification and statistical analysis

The perovskite solar cells’ cross-sectional morphologies were characterised using a scanning electron microscope (SEM and FEI Sirion-200). Photovoltaic data was collected from WACOM AAA simulator connected I-V software. The Incident Photon-to-electron Conversion Efficiency (IPCE) measurement was carried out on a BENTHAM PVE300 under a 300–800 nm wavelength, using a tungsten halogen lamp source. Figures were produced by Origin 9 from the raw data. IR images were captured with an FLIR T425 camera positioned at 10 mm.

## Data Availability

• All data reported in this paper will be shared by the lead contact upon reasonable request. • No new code was generated during the course of this study. • Any additional information required to reanalyze the data reported in this paper is available from the lead contact upon reasonable request.
