# Supplementary material for: Certified high-efficiency “large-area” perovskite solar module for Fresnel lens-based concentrated photovoltaics
Source: iScience. 2023 Feb 2;26(3):106079. doi: 10.1016/j.isci.2023.106079 (PMC9950384; doi:10.1016/j.isci.2023.106079)
Supplement: Document S1. Figures S1–S7 and Table S1 [file mmc1.pdf]

iScience, Volume *26*

Supplemental information

Certified high-efficiency “large-area” perovskite solar module for Fresnel lens-based concentrated photovoltaics

Anurag Roy, Bin Ding, Maria Khalid, Mussad Alzahrani, Yong Ding, Asif A. Tahir, Senthilarasu Sundaram, Sachin Kinge, Abdullah M. Asiri, Andre Slonopas, Paul J. Dyson, Mohammad Khaja Nazeeruddin, and Tapas K. Mallick

Supplemental Information

Certified high-efficiency "large-area" perovskite solar cells module for Fresnel lens-based concentrated photovoltaic

Anurag Roy,[1,#,*] Bin Ding,[2,#] Maria Khalid,[1] Mussad Alzahrani,[3,] Yong Ding,[2,4] Asif A. Tahir,[1] Senthilarasu Sundaram,[5] Sachin Kinge,[6] Abdullah M. Asiri,[7] Andre Slonopas,[8] Paul J. Dyson,[2,] * Mohammad Khaja Nazeeruddin,[2,] * Tapas K. Mallick[1,*]

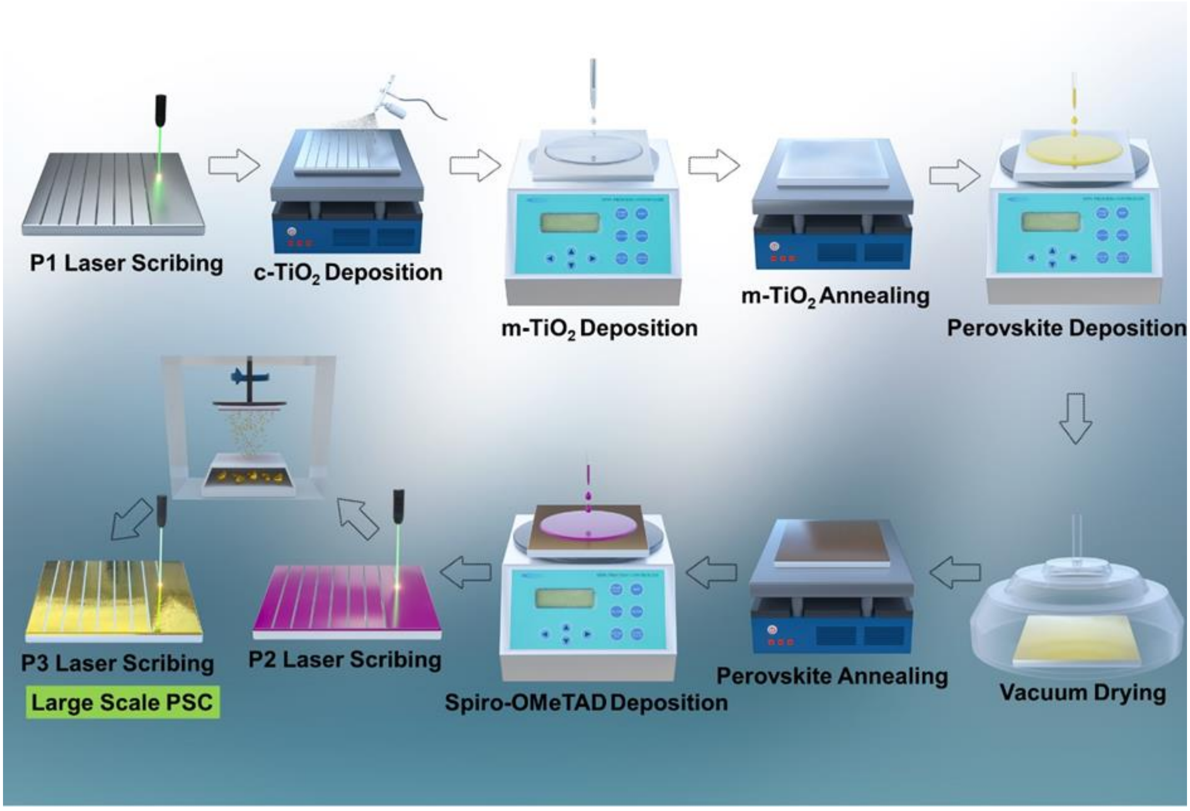

Figure S1. **Stepwise schematic illustration of "large area" perovskite solar module fabrication, Related to STAR Methods.**

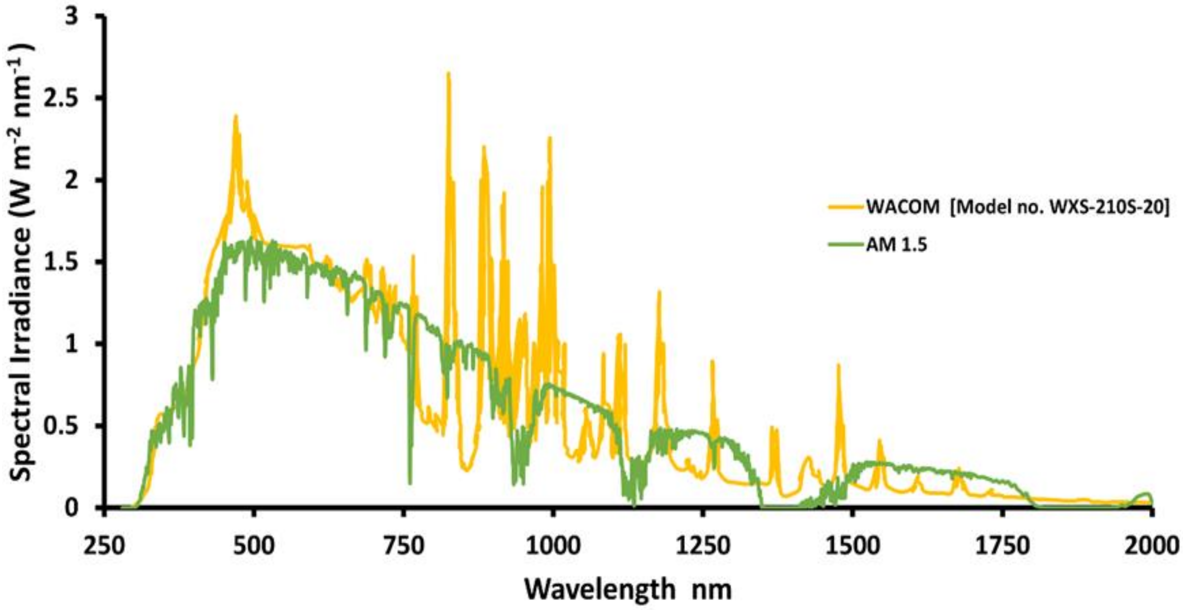

Figure S2. **Solar Spectral Irradiance of WACOM [Model no. WXS-210S-20] and the actual solar irradiance both for AM 1.5, Related to Figure 2.**

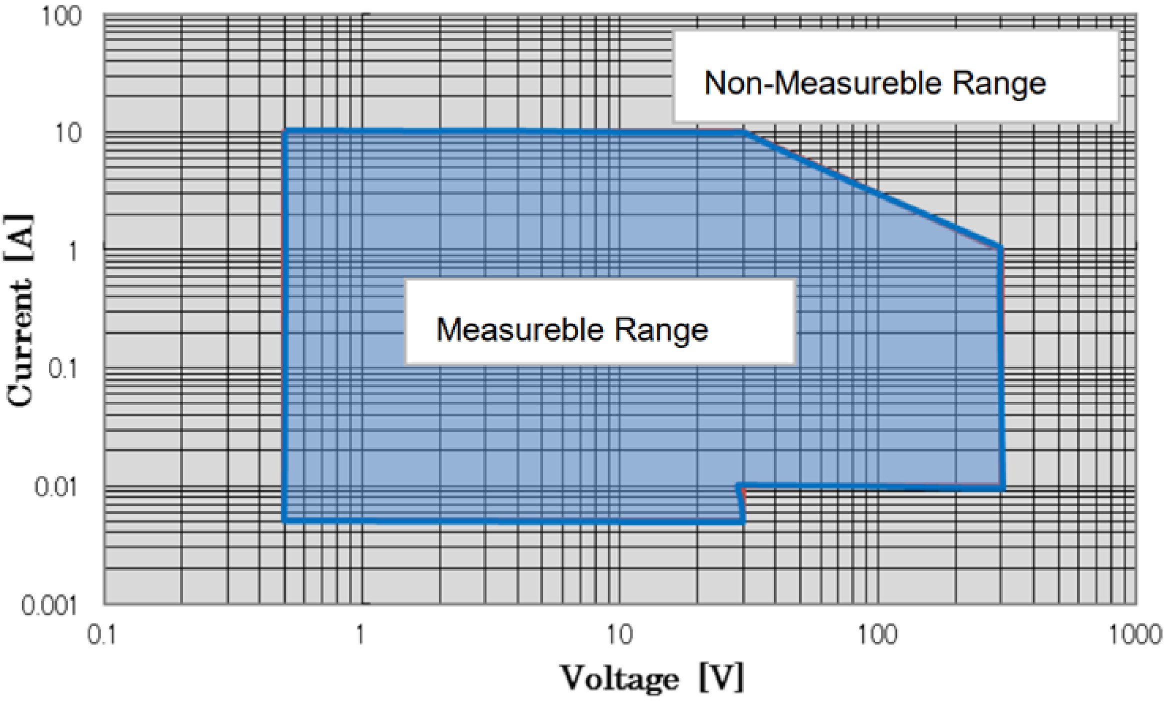

Figure S3. Current-volatge measurable and non-measurable operating range of EKO: MP-160, Related to Figure 2 and STAR Methods.

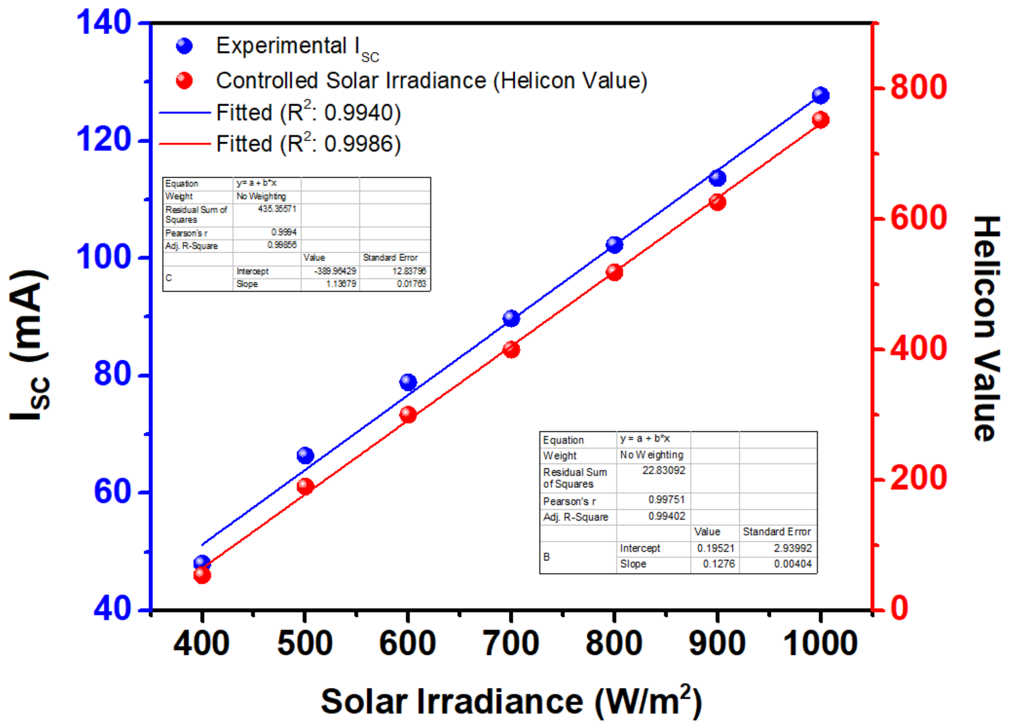

Figure S4. Solar simulator calibration plot for different solar irradiances by controlling the Helicon value, Related to Figure 3, Figure 4 and Figure 5.

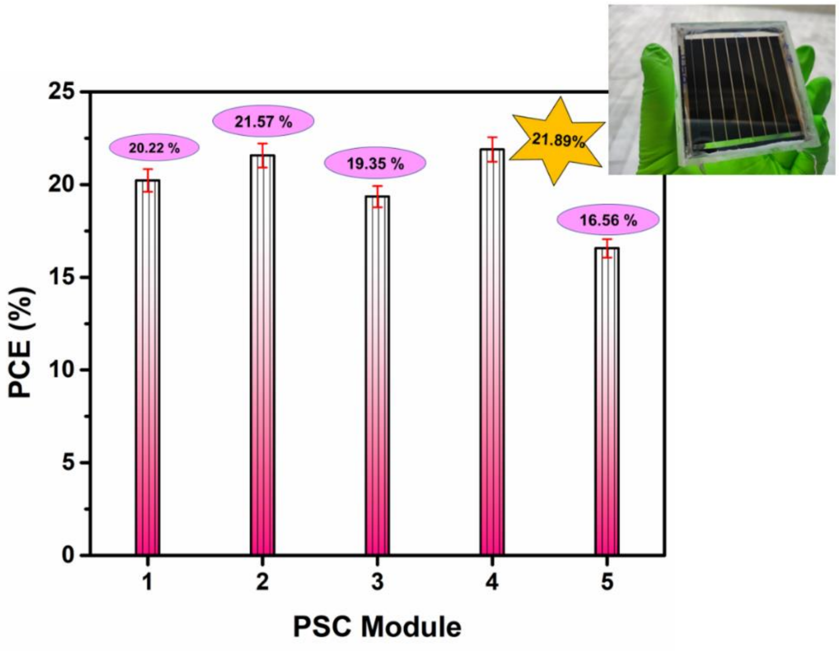

Figure S5. Comparative PCE was recorded for five PSC modules, where module number 4 (inset: corresponding photograph) was considered for champion PCE and selected for further experiment, Related to Figure 1A.

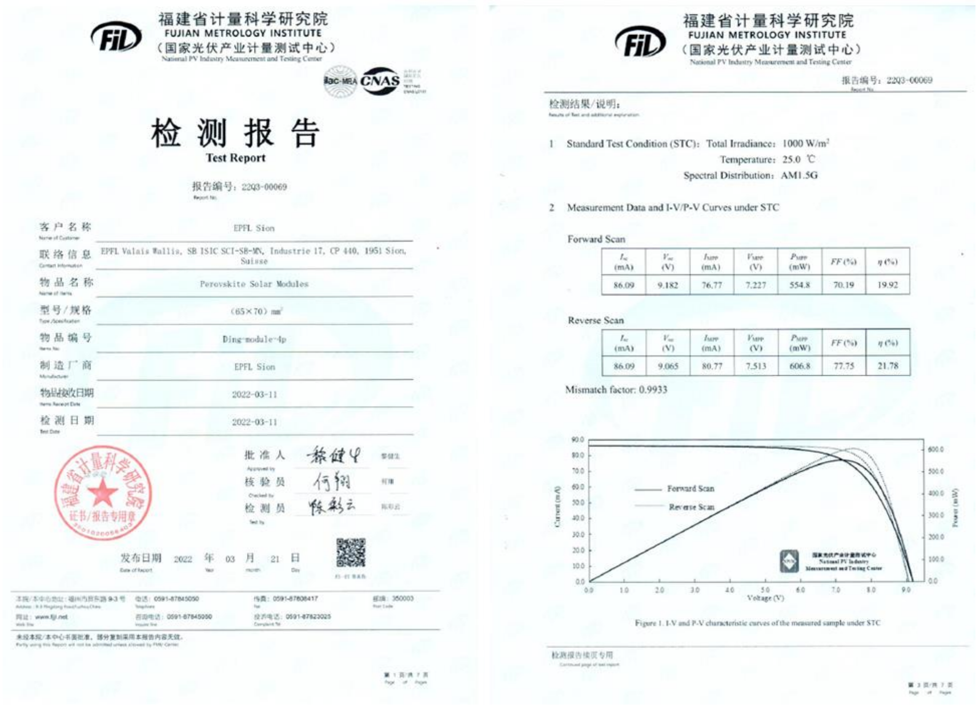

福建省计量科学研究院
FUJIAN METROLOGY INSTITUTE
（国家光伏产业计量测试中心）
National PV Industry Measurement and Testing Center

检 测 报 告
Test Report

报告编号：22Q3-00069
Report No.

客户名称 Name of Customer	EPFL Sion
联络信息 Contact Information	EPFL Valais Wallis, SB ISIC SCI-SB-MN, Industrie 17, CP 440, 1951 Sion, Suisse
物品名称 Name of Items	Perovskite Solar Modules
型号/规格 Type /Specification	（65×70） mm²
物品编号 Items No.	Ding-module-4p
制造厂商 Manufacturer	EPFL Sion
物品接收日期 Items Receipt Date	2022-03-11
检测日期 Test Date	2022-03-11

批准人 Approved by
核验员 Checked by
检测员 Test by

发布日期 Date of Report 2022 年 03 月 21 日

福建省计量科学研究院
FUJIAN METROLOGY INSTITUTE
（国家光伏产业计量测试中心）
National PV Industry Measurement and Testing Center

报告编号：22Q3-00069

检测结果/说明：

1 Standard Test Condition (STC)：Total Irradiance：1000 W/m²
Temperature：25.0 ℃
Spectral Distribution：AM1.5G

2 Measurement Data and I-V/P-V Curves under STC

Forward Scan

I_{sc} (mA)	V_{oc} (V)	I_{MPP} (mA)	V_{MPP} (V)	P_{MPP} (mW)	FF (%)	η (%)
86.09	9.182	76.77	7.227	554.8	70.19	19.92

Reverse Scan

I_{sc} (mA)	V_{oc} (V)	I_{MPP} (mA)	V_{MPP} (V)	P_{MPP} (mW)	FF (%)	η (%)
86.09	9.065	80.77	7.513	606.8	77.75	21.78

Mismatch factor: 0.9933

Figure 1. I-V and P-V characteristic curves of the measured sample under STC

Figure S6. The certified result of the "large area" perovskite solar cell measured at Fujian Metrology Institute, Related to Figure 1A.

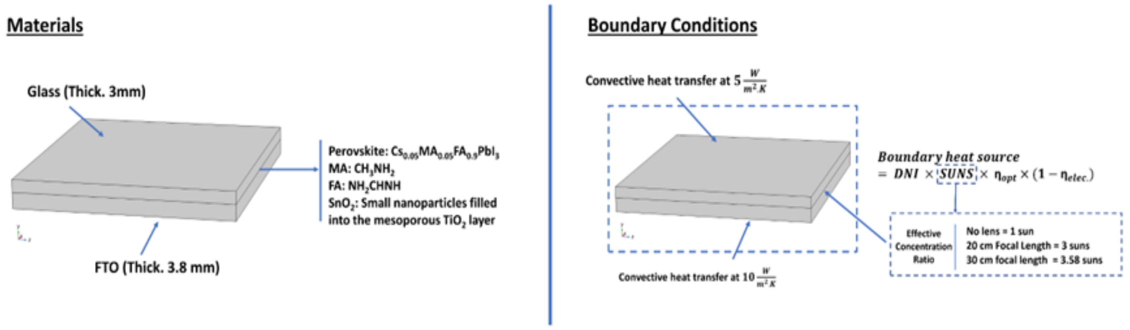

Governing Equation

The heat transfer rate in the PSC is governed by considering the energy conservation law for the transient state condition

$$DNI \times Sun \times eta_{optic} \times (1 - eta_{\ electric}) = \nabla\left[\left(-\frac{L}{K}\left(\frac{\Delta T}{\partial t}\right) - h\left(T_{surface} - T_{surrounding}\right) - \varepsilon\,\sigma\left(T^4_{surface} - T^4_{surrounding}\right)\right]$$

Symbol	Definition /Unit
DNI	Direct Normal Irradiance ($\frac{W}{m^2}$).
Sun	Effective concentration ratio (sun = $\frac{W}{m^2}$).
eta_{optic}	Optical Efficiency (%)
$eta_{electric}$	PSC electrical efficiency (%)
L	PSC layer thickness (m)
K	Thermal conductivity (W/(m.K))
h	Convective Heat Transfer Coefficient ($\frac{W}{m^2.K}$)
ΔT	Temperature gradient (°C)
ε	Emissivity (-)
σ	Stefan–Boltzmann constant ($5.67E - 8\ \frac{W}{m^2.K^4}$)

Figure S7. COMSOL heat transfer modelling boundary condition employed to the PSC when optics were included, and COMSOL simulation results of the surface and interfaced temperature of the module, Related to Figure 7A.

**Table S1. Stabilized PV parameters of the Fresnel lens-integrated PSC module (FL-PSC) under different solar irradiance at a lens-to-cell distance of 10, 20 and 30 cm, Related to Figure 5A-5F.**						
**Solar Irradiation (mW/cm^2)**	**I_{SC} (mA)**	**V_{OC} (mV)**	**FF ± 0.2**	**PCE ± 0.01 (%)**	**Power (mW)**	**Power Enhancement**
**Only PSC**						
400	27.88	8735.23	0.78	16.61	189.96	69.21 %
500	39.78	8832.73	0.77	19.13	273.41	
600	47.96	8946.73	0.77	19.65	337.11	
700	54.84	8958.16	0.76	18.72	374.42	
800	62.82	9029.83	0.77	19.18	438.57	
900	70.66	9083.62	0.77	19.29	496.26	
1000	81.67	9237.49	0.82	21.89	617.08	
**FL-PSC system at a lens to cell distance of 10 cm**						
400	44.27	8681.94	0.72	15.31	278.09	67.24 %
500	63.72	8598.83	0.72	17.35	397.33	
600	76.80	8654.25	0.72	17.71	486.51	
700	88.12	8671.08	0.73	17.29	556.08	
800	101.57	8729.80	0.72	17.28	638.91	
900	113.83	8778.33	0.72	17.42	721.98	
1000	125.05	9174.40	0.74	20.73	849.02	
**FL-PSC system at a lens to cell distance of 20 cm**						
400	84.97	8417.70	0.69	15.80	550.65	67.99 %
500	121.73	8629.79	0.68	16.02	700.78	
600	146.25	8760.40	0.69	17.04	891.09	
700	168.03	8957.85	0.69	17.03	1044.31	
800	191.94	8961.34	0.68	18.39	1284.85	
900	216.78	9037.54	0.67	19.37	1528.88	
1000	242.53	9380.52	0.70	20.27	1720.38	
**FL-PSC system at a lens to cell distance of 30 cm**						
400	102.95	8771.34	0.72	16.13	680.90	76.37%
500	141.56	8925.87	0.70	17.12	870.71	
600	172.84	8969.59	0.69	17.01	1050.72	
700	196.87	8991.64	0.70	17.35	1246.52	
800	222.54	8950.11	0.72	17.90	1450.19	
900	251.28	8948.35	0.71	18.71	1711.44	
1000	502.78	9390.24	0.61	16.38	2882.16	
